# From data to decision: Scaling artificial intelligence with informatics for epilepsy management

**DOI:** 10.1002/ctm2.70108

**Published:** 2024-12-13

**Authors:** Nishant Sinha, Alfredo Lucas, Kathryn Adamiak Davis

**Affiliations:** ^1^ Department of Neurology University of Pennsylvania Philadelphia Pennsylvania USA

The integration of artificial intelligence (AI) into epilepsy research presents a critical opportunity to revolutionize the management of this complex neurological disorder.^1^ Despite significant advancements in developing AI algorithms to diagnose and manage epilepsy, their translation into clinical practice remains limited. This gap underscores the urgent need for scalable AI and neuroinformatics approaches that can bridge the divide between research and real‐world application.^2^ The ability to generalize AI models from controlled research environments to diverse clinical settings is crucial. Current efforts have made substantial progress, but they also reveal common pitfalls, such as overestimation of model performance due to data leakage and the challenges of small sample sizes, which hinder the generalization of these models.

To address these challenges and fully realize the potential of AI in epilepsy care, a robust framework for data sharing and collaboration across research centres is essential. Cloud‐based informatics platforms offer a promising solution by enabling the aggregation and harmonization of large, multisite datasets. These platforms can facilitate the development of AI models that are not only powerful but also scalable and generalizable across different patient populations and clinical scenarios. In this commentary, we will explore the common methodological errors that lead to overly optimistic AI models in epilepsy research and propose strategies to overcome these issues. We will also discuss the importance of collaborative data sharing in building robust, clinically relevant AI tools and highlight the role of advanced neuroinformatics infrastructures in supporting the translational pathway from research to clinical practice (Figure 1).

**FIGURE 1:**
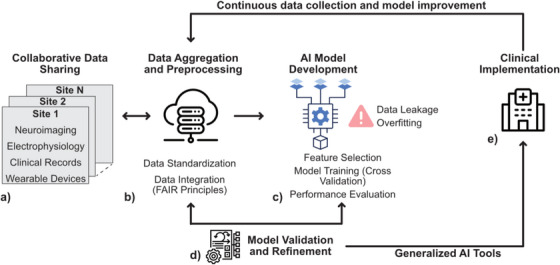
Artificial intelligence (AI)‐powered epilepsy management. (a) Collaborative sharing of epilepsy data is critical to developing AI models that can be generalized for clinical implementation. Epilepsy data broadly includes multimodal neuroimaging, electrophysiology, medical information from electronic health records, and wearable device data. (b) Neuroinformatics infrastructures provide the technological foundation essential to securely aggregate, manage, and analyze large‐scale epilepsy datasets. When selecting an infrastructure, researchers should prioritize those that adhere to findability, accessibility, interoperability, and reusability (FAIR) principles. (c) AI model development includes feature selection, model training, cross‐validation, and performance evaluation while taking precautions against data leakage and overfitting. (d) Model validation and refinement is an iterative process that improves AI models through shared data and feedback. (e) Clinical implementation translates AI research into clinical practice, with a feedback loop to ensure continuous data collection and model improvement.

## 1 ADDRESSING METHODOLOGICAL PITFALLS IN AI DEVELOPMENT

The promise of AI in epilepsy research is often hampered by methodological errors that lead to overly optimistic performance metrics. One of the most significant issues is *data leakage*, which occurs when information from outside the training dataset influences the model, resulting in an overestimation of its predictive power. This can happen when features are derived from the entire dataset rather than just the training subset.^3^ To mitigate this, strict separation between training and test datasets is essential and feature selection must be performed within each fold of the cross‐validation process independently. Nested cross‐validation, where model selection and performance estimation are conducted separately, further reduces the risk of data leakage.

Another common error is the *improper application of cross‐validation* techniques. Often, researchers perform feature selection or hyperparameter tuning on the entire dataset before cross‐validation, leading to inflated performance metrics. The correct approach is to embed these steps within each fold of the cross‐validation process to ensure that the test data remain completely unseen until the final evaluation. This practice helps prevent overfitting and provides a more accurate estimate of how the model will perform on new data.

*Small sample size* presents a third challenge, particularly in epilepsy research, where datasets are often of modest size and heterogeneous. Small datasets can lead to overfitting, where the model learns patterns specific to the training data but fails to generalize to new data. Addressing this requires both methodological rigour and collaborative efforts to pool data across multiple sites, thereby creating larger, more diverse datasets. Data augmentation techniques, such as generating synthetic data, can also help increase the effective size of the training set.

## 2 THE POWER OF COLLABORATIVE DATA SHARING

The development of robust AI models in epilepsy is further strengthened by collaborative data sharing, which allows researchers to pool datasets from multiple sources, increasing both the size and diversity of the data available for training. Epilepsy is a highly heterogeneous disorder, and individual research centres often have access to only small modest‐size cohorts. By aggregating data across different sites, researchers can develop AI tools that are more representative of the broad clinical reality to improve generalizability and reliability across diverse clinical settings.

Collaborative data sharing also enables the replication of studies, which is critical for validating AI models across different cohorts to ensure that the models are both accurate and reproducible. Such collaboration fosters the sharing of expertise and resources, allowing researchers to tackle complex challenges, such as integrating multimodal data—neuroimaging, electrophysiology and clinical records—into more sophisticated AI models.

## 3 ROLE OF NEUROINFORMATICS INFRASTRUCTURES

To support effective data sharing and utilization across multiple sites, advanced neuroinformatics infrastructures are indispensable. Platforms like EBRAINS, Pennsieve (https://app.pennsieve.io/) and OpenNeuro, among others, provide the technological foundation needed to securely aggregate, manage and analyze large‐scale epilepsy datasets.^4^, ^5^ These platforms enable researchers to apply standardized methods and tools across different datasets to ensure the rigour, robustness and reproducibility of AI models.

Neuroinformatics platforms also adhere to the principles of making data findable, accessible, interoperable and reusable, which is crucial for effective data sharing.^6^ By facilitating data harmonization and integration, these platforms ensure that data from multiple sources can be combined and analyzed consistently.^7^ Furthermore, neuroinformatics infrastructures support collaborative analysis by allowing researchers to share not just data, but also the algorithms and models developed from that data. For example, researchers could share their electrode localization outputs generated from a standardized pipeline,^8^ together with their intracranial electroencephalography recordings, and the deep learning model trained for seizure detection. Alternatively, researchers might only share their data,^9^ and the preprocessing and model building could all happen within these infrastructures.^10^ This fosters an open science environment where AI models can be tested and refined across different datasets to accelerate the development of clinically applicable tools.

## 4 CONCLUSION

In summary, the advancement of AI in epilepsy research depends on both methodological rigour and collaborative efforts. By addressing common errors in AI model development and leveraging the power of collaborative data sharing, we can build robust, clinically relevant tools. Neuroinformatics infrastructures provide the necessary support for these endeavours to ensure that AI models are not only powerful but also applicable in real‐world clinical settings. These combined strategies are essential to translate AI research into tangible improvements in epilepsy care, ultimately leading to better patient outcomes.

## AUTHOR CONTRIBUTION

*Conceptualization*: Nishant Sinha, Alfredo Lucas, Kathryn Adamiak Davis. *Writing—original draft preparation and revision for intellectual content*: Nishant Sinha, Alfredo Lucas, Kathryn Adamiak Davis.

## ETHICS STATEMENT

The authors declare no conflict of interest.
